# Late-Relapsing Hepatitis after Yellow Fever

**DOI:** 10.3390/v12020222

**Published:** 2020-02-17

**Authors:** Izabela Maurício Rezende, Leonardo Soares Pereira, Jordana Rodrigues Barbosa Fradico, Marcelo Antônio Pascoal Xavier, Pedro Augusto Alves, Ana Carolina Campi-Azevedo, Elaine Speziali, Lívia Zignago Moreira dos Santos, Natalia Soares Albuquerque, Indiara Penido, Tayrine Araujo Santos, Ana Paula Dinis Ano Bom, Andrea Marques Vieira da Silva, Camilla Bayma Fernandes, Carlos Eduardo Calzavara, Erna Geessien Kroon, Olindo Assis Martins-Filho, Andréa Teixeira-Carvalho, Betânia Paiva Drumond

**Affiliations:** 1 Laboratório de Vírus, Instituto de Ciências Biológicas, Universidade Federal de Minas Gerais, Minas Gerais 31270-901, Brazil; izabelamauriciorezende@gmail.com (I.M.R.); ernagkroon@gmail.com (E.G.K.); 2 Eduardo de Menezes Hospital, Minas Gerais 30622-020, Brazil; leosoaresgallo@yahoo.com.br (L.S.P.); lizms@msn.com (L.Z.M.d.S.); albuquerque.natalia@gmail.com (N.S.A.); indypenido@hotmail.com (I.P.); tayrine.araujo@fhemig.mg.gov.br (T.A.S.); 3 Grupo Integrado de Pesquisas em Biomarcadores, Instituto René Rachou, Fundação Oswaldo Cruz/FIOCRUZ, Minas Gerais 30190-002, Brazil; jordanafradico@gmail.com (J.R.B.F.); campiazevedo@gmail.com (A.C.C.-A.); fariaspeziali@gmail.com (E.S.); oamfilho@gmail.com (O.A.M.-F.); atcteixeira@gmail.com (A.T.-C.); 4 Departamento de Anatomia Patológica e Medicina Legal, Faculdade de Menincina, Universidade Federal de Minas Gerais, Minas Gerais 31270-901, Brazil; mpascoal@medicina.ufmg.br; 5 Imunologia de Doenças Virais, Instituto René Rachou, Fundação Oswaldo Cruz/FIOCRUZ, Minas Gerais 30190-002, Brazil; pedroaugustoalves@yahoo.com.br; 6 Laboratório de Tecnologia Imunológica, Bio-Manguinhos, Fundação Oswaldo Cruz/FIOCRUZ, Rio de Janeiro 21040-900, Brazil; 7 Imunologia Celular e Molecular, Instituto René Rachou, Fundação Oswaldo Cruz/FIOCRUZ, Minas Gerais 30190-002, Brazil; calzavara@gmail.com

**Keywords:** yellow fever virus, yellow fever, liver damage, viral persistence, hepatitis, liver biopsy, persistent infection, relapsing hepatitis, emerging virus, flavivirus

## Abstract

One patient presented hyporexia, asthenia, adynamia, and jaundice two months after acute yellow fever (YF) onset; plus laboratory tests indicating hepatic cytolysis and a rebound of alanine and aspartate transaminases, and total and direct bilirubin levels. Laboratory tests discarded autoimmune hepatitis, inflammatory or metabolic liver disease, and new infections caused by hepatotropic agents. Anti-YFV IgM, IgG and neutralizing antibodies were detected in different times, but no viremia. A liver biopsy was collected three months after YF onset and tested positive for YFV antigens and wild-type YFV-RNA (364 RNA-copies/gram/liver). Transaminases and bilirubin levels remained elevated for five months, and the arresting of symptoms persisted for six months after the acute YF onset. Several serum chemokines, cytokines, and growth factors were measured. A similar immune response profile was observed in the earlier phases of the disease, followed by more pronounced changes in the later stages, when transaminases levels returned to normal. The results indicated viral persistence in the liver and continual liver cell damage three months after YF onset and reinforced the need for extended follow-ups of YF patients. Further studies to investigate the role of possible viral persistence and the immune response causing relapsing hepatitis following YF are also necessary.

## 1. Introduction

Despite intense study, relatively little is known about yellow fever (YF) in humans beyond descriptive records [1]. YF is classically characterized by three periods: infection, remission, and intoxication, with a rise in serum transaminase levels. Aspartate aminotransferase (AST) levels are usually higher than alanine aminotransferase (ALT), reflecting disease severity [1,2,3]. After the acute phase, the duration of jaundice is unknown [1]. Weakness and fatigue may last several weeks [1] and slightly abnormal liver function may persist for 60 days or more [1,2,4]. Here, we present an investigation of one case of symptomatic late-relapsing hepatitis after acute yellow fever virus (YFV) infection.

## 2. Materials and Methods 

During a YF outbreak in Brazil, one patient had the onset of fever, headache, epigastric pain, hyporexia, myalgia, nausea, red diuresis, and mild dyspnea in January/5/2017. He looked for medical care at a local hospital (Caratinga/Minas Gerais) at the fourth day post-symptoms onset (DPS). Laboratory tests demonstrated highly increased levels of AST = 4560 IU/L (75.5 µkat/L) (normal range: 10–40 IU/L) and ALT = 3710 IU/L (61.59 µkat/L) (normal range: 10–40 IU/L) along with thrombocytopenia, and elevated levels of urea and creatinine.

The patient was referred to Eduardo de Menezes Hospital (HEM) (Belo Horizonte/Minas Gerais). Routine tests for clinical parameters, diagnosis of infectious diseases (File S1), and imaging exams (x-ray, computed tomography, and colangio magnetic resonance) were run. The patient was hospitalized and monitored for signs, symptoms and laboratory abnormalities related to renal and liver function, and blood coagulation (AST, ALT, alkaline phosphatase (APhos), gamma-glutamyltransferase (GGT), total bilirubin (TBil), direct bilirubin (DBil), urea, creatinine (Cr), creatine kinase (CK), prothrombin and complete blood cell count) (File S1).

By the time the patient returned with jaundice and complaining of postprandial fullness (78th DPS), he was rehospitalized at HEM. The patient was clinically evaluated and monitored (signs, symptoms, and laboratory abnormalities: renal, liver functions, imaging exams) (File S1). Markers for autoimmune hepatitis, metabolic liver disease, or infections caused by hepatotropic infectious agents were evaluated (File S1). Urine or sera were used in molecular or serological tests to diagnose infectious diseases [5,6,7,8,9] (File S1). A liver biopsy was collected (93rd DPS) and submitted to histological and immunohistochemistry analyzes, plus qualitative and quantitative molecular investigation of YFV RNA [5,9] (File S1). Several serum chemokines, cytokines, and growth factors were measured in sera collected at the 36th, 78th, 197th, and 306th DPS (File S1). Sera were also used to investigate anti-YFV IgM (immunochromatographic test), anti-YFV IgG (in house ELISA) and anti-YFV neutralizing antibodies (plaque reduction neutralization test: PRNT) [10] (File S1).

The study was authorised by the Minas Gerais state Health Department and approved by the Ethics Committee on Human Research at René Rachou Institute/FIOCRUZ (CAAE 65814417.0.0000.5091 and CAAE: 43000815.7.0000.5091). The subjects provided informed consent to the research.

## 3. Results

A 51-year-old male rural worker suspected of YF was hospitalized at the sixth DPS at HEM. The patient, a former alcoholic and smoker, had systemic arterial hypertension and reported no history of YF vaccination. By the time he was first admitted at HEM, he presented myalgia, asthenia, inappetence, vomiting, moderate jaundice and intense dehydration; but no bleeding, diuresis reduction, or signs/symptoms of hepatic encephalopathy. He presented a body temperature of 37 °C, heart rate of 84 beats per minute and, blood pressure of 150/100 mm Hg. Abdominal examination showed tenderness in the right upper quadrant, but no rebound tenderness, Murphy’s sign was absent, and a Glasgow Coma Scale of 15 was reported.

On the next day, the patient had jaundice and abnormal levels of AST, ALT along with elevated levels of APhos, GGT, TBil, DBil, urea, and Cr (Table 1). Low levels of CK, leucopenia, thrombocytopenia, and lymphopenia were observed (Table 1, Table S1). The patient showed elevated blood pressure controlled with antihypertensive therapy (losartan), and an unremarkable chest radiograph. In the following days, he presented a progressive improvement of his clinical condition, hepatic, and renal functions (Table 1, Table S1). The hospital discharge occurred at 14th DPS when jaundice ameliorated (Table 1), and the patient was advised to return within two weeks. 

By the time the patient had hospital discharge, YF diagnosis was only based on the patient´s clinical picture. Since the patient lived in an area with an ongoing YF outbreak, he was advised and vaccinated with the 17DD-YF vaccine (Bio-Manguinhos, FIOCRUZ), on the next day (15th DPS) after hospital discharge. Routine diagnostics tests run by State Reference Laboratory (FUNED-MG) later confirmed YF infection by detection of anti-YFV IgM and viral isolation followed by indirect immunofluorescent test, and anti-DENV IgM non-reactant (Table 1). 

At the 36th DPS, the patient returned to HEM reporting sporadic events of postprandial fullness and epigastric pain. The laboratory liver tests indicated the persistence of elevated AST, ALT, APhos (similar levels to those observed at hospital discharge), GGT, TBil, DBil, urea and, Cr (Table 1). The general clinical profile of the patient was good, without jaundice (Table 1, Table S1), and he was advised to return within one month. At the 64th DPS, he looked for medical care in a local hospital, reporting hyporexia, postprandial fullness, malaise, and jaundice. Laboratory tests indicated liver injury, with a pronounced increase in AST, ALT, TBil, and DBil levels (Table 1). At the 70th DPS, he was hospitalized in a local hospital (Caratinga). Since jaundice persisted along with discrete non-specific hepatomegaly revealed by computed tomography, the patient was transferred to HEM, at the 78th DPS. During the time the patient was rehospitalized at HEM (78th–96th DPS), he presented postprandial fullness and persisting jaundice, altered ALT, AST, APhos, GGT, TBil, and DBil tests (Table 1). Imaging exams (computed tomography) indicated a discrete non-specific mild hepatomegaly and discarded biliary obstruction (Table 1). Since the patient had an improvement in clinical profile, without fever, with ALT and AST bellow 1000 IU/L (16.6 µkat/L), reduced levels of TBil and DBil, stable and normal ratio of prothrombin time (Table 1, Table S1), without bleeding or encephalopathy, he was discharged at the 96th DPS, and he was advised to return within 15 days.

When the patient was rehospitalized at HEM, his serum and urine (78th DPS) were tested for the presence of YFV and were negative. His serum was tested to investigate infection caused by the DENV, CHIKV, Human immunodeficiency virus, Hepatitis A virus, Hepatitis C virus, Hepatitis B virus, Epstein Barr virus, and *Toxoplasma gondii*, and the results were negative (Table 1). Markers for autoimmune hepatitis were investigated and negative (Table 1). Increased erythrocyte sedimentation rate and levels of ferritin were observed (Table 1) and no Kayser–Fleischer ring was observed at ophthalmological evaluation.

**Figure 1:**
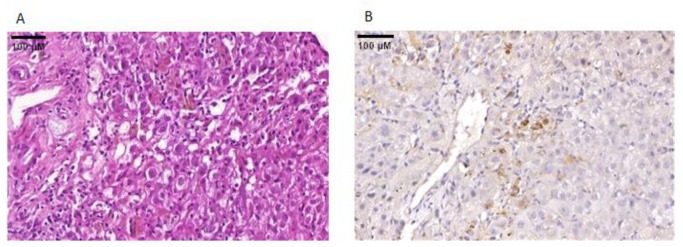
Detection of YFV antigens in the liver biopsy collected at the 93rd day post-onset of first symptoms of yellow fever. (**A**) Histopathology of the hepatic biopsy. Portal and lobular mononuclear infiltrate, predominantly in zones 2 and 3 of the hepatic acini, and hepatocyte vacuolar degeneration are observed (H&E, 40X); (**B**) Immunohistochemistry of the hepatic biopsy. The yellow fever antigen was detected by immunohistochemistry in the cytoplasm of hepatocytes (brown areas) (DAB [3, 3’-diaminobenzidine], using a YFV-specific monoclonal antibody; 40X). YFV-specific monoclonal antibody was kindly provided by Dr. Pedro Fernando da Costa Vasconcelos, from Evandro Chagas Institute, Pará, Brazil).

A liver biopsy procedure was performed at the 93rd DPS, and histological analyzes showed portal and lobular mononuclear infiltrate, predominantly in zones 2 and 3 of the hepatic acini, and vacuolar degeneration (Figure 1A). Immunohistochemistry analyzes demonstrated hepatic reactivity to anti-YFV in small foci, mostly at zones 2 and 3 of the hepatic acini (Figure 1B).

**Figure 2:**
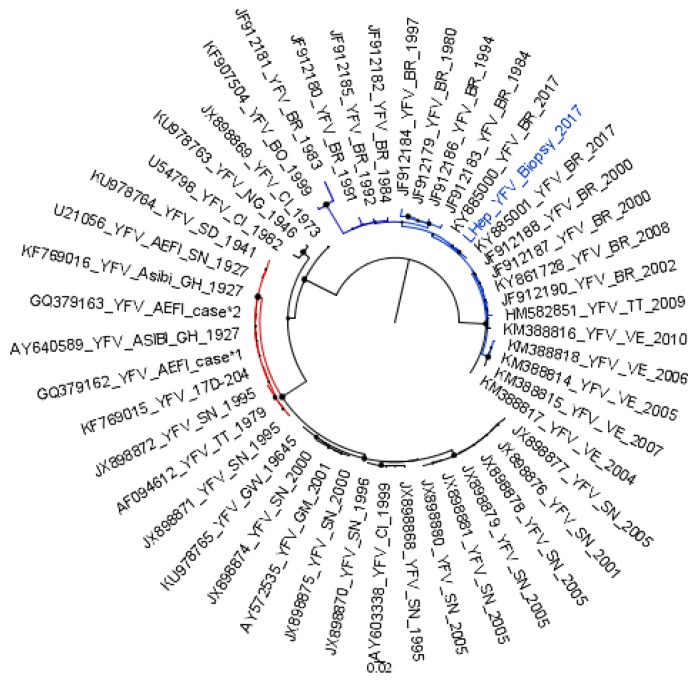
Detection and phylogeny analyzis of YFV RNA sequences in the liver biopsy collected at the 93rd day post-onset of first symptoms of yellow fever. A sub-tree from the maximum-likelihood tree inferred using Yellow fever virus sequences (211 nucleotides) is shown. YFV strains from South American genotypes are shown in blue. The YFV detected in the liver biopsy (highlighted in blue) is grouped within the South American clade, apart from the vaccine strains and vaccine-related strains (red). The YFV strains belonging to African genotypes are shown in black, except vaccine and vaccine-related strains, which are colored in red. YFV strains are identified by their Genbank identification number, and by the year they were detected. The bootstrap values are represented by circles drawn in scale in the nodes. The scale indicates nucleotide substitution per site. The tree was reconstructed using the Kimura-2 parameters nucleotide substitution model with gamma distribution (four categories), and analyzes were performed using MEGA7 [12].

A fragment of the liver biopsy was used to investigate the presence of YFV RNA. The sample tested positive for the presence of YFV RNA by qualitative and quantitative RT-qPCR (364 genomic copies/gram/liver). Nucleotide sequencing phylogenetic analyzis (based on partial NS5 sequence) indicated the virus clustering apart from vaccine strains, within the South American genotypes, demonstrating the presence of wild-type YFV RNA in the liver biopsy (Figure 2).

**Figure 3:**
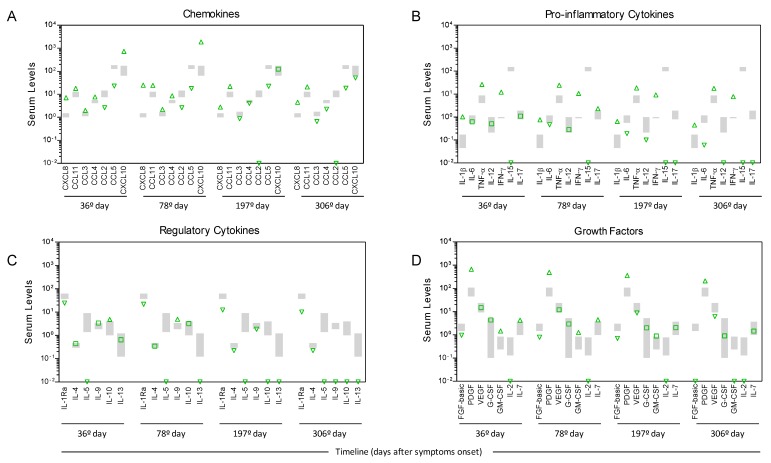
Kinetics of serum biomarkers of the immune response in a Yellow fever patient compared to the healthy control group. (**A**) Chemokine levels (CXCL8, CCL11, CCL3, CCL4, CCL2, CCL-5, and CXCL10); (**B**) Pro-inflammatory cytokine levels (IL1-β, IL-6, TNF-α, IL-12, IFN-ɣ, IL-15 and IL-17); (**C**) Regulatory cytokine levels (IL-1Ra, IL-4, IL-5, IL-9, IL-10 and IL-13); (**D**) Growth factors levels (FGF-basic, PDGF, VEGF, G-CSF, GM-CSF, IL-2 and IL-7) were measured at the 36th, 78th, 197th and 306th day post-symptom onset by high-performance microbeads 27-plex assay, as described in File S1. Green arrows represent increased (up) or decreased (down) levels of serum biomarkers in comparison to the 95% confidence interval (CI) of the mean values found in the reference control group (*n* = 16) underscored by gray boxes.

The analyzis of the serum biomarkers of immune response revealed a massive increase in chemokines (CXCL8, CCL11, CCL3, CCL4, and CXCL10) and pro-inflammatory cytokines (IL-1β, TNF-α and IFN-ɣ) besides enhanced levels of the regulatory cytokine (IL-10) and growth factors (PDGF, GM-CSF and IL-7) at the 36th day post-symptom onset when compared to the levels observed for healthy controls. Moreover, lower levels of CCL2 and CCL5, IL-15, IL-1Ra, IL-5, FGF-basic, and IL-2 were observed in the YF-infected patients in comparison to the control group (Figure 3). The levels of most chemokines, pro-inflammatory cytokines, regulatory cytokines, and growth factors remained elevated for 78 days after the onset of symptoms, with a slight increase in the CXCL8, CXCL10, and IL-17 levels along with a decrease in the IL-6, IL-12, and IL-13 levels. Conversely, lower levels of CXCL8, CCL3, CCL4, CCL2, CXCL10, IL-6, IL-12, and IL-17, as well as an important reduction in IL-10 and IL-13 levels, were observed at the 197th DPS compared to previous samples collected at the 36th and the 78th DPS. Regardless of the increase in the CXCL8, decreasing levels of CCL3, CCL4, CXCL10, and IL-6, together with an important reduction in the IL-10, IL-13, FGF-basic and GM-CSF levels were observed at the 306th DPS compared to previous samples collected at the 36th, 78th, and 197th DPS (Figure 3). 

Sera collected at the 8th, 78th, 83rd, and 306th DPS were positive for the presence of IgM. Sera collected at the 8th, 36th, 78th, 83rd, 197th, and 306th DPS were positive for the presence of anti-YFV IgG (Figure S1). Sera collected at the 78th and 83rd DPS also tested positive for the presence of neutralizing antibodies: PRNT 80% up to sera dilution 1:80). 

After 30 days since medical discharge from the second hospitalization (124th DPS), the patient reported hyporexia, asthenia, and adynamia; but no jaundice. Since laboratory tests indicated a significant decrease in AST, ALT, APhos, GGT, TBil, and DBil levels, the patient was discharged and advised to return within 45 days (Table 1). Liver tests run at the 186th DPS in a local hospital indicated normal or slightly elevated levels of liver injury markers (Table 1). He returned to HEM (197th DPS) and reported the persistence of symptoms and difficulties to work, but presented normal levels of AST and ALT. The patient was released, returned at the 306th DPS, and no longer reported asthenia or adynamia, and liver tests were normal. Given the improvement of clinical status (Table 1, Table S1), the patient was finally discharged.

During the whole time the patient was attended, he remained with hemodynamic stability and normal mental status; however, no observations of cardiovascular arrhythmia, respiratory abnormalities, ascites, or urinary or bowel habits changes were reported. During the same period, the patient denied the use of alcohol or natural teas, and only reported the use of losartan.

## 4. Discussion

Here we report a case of a YF patient with the typical acute phase of YF, followed by late-relapsing hepatitis. This case was characterized by higher levels of ALT compared to AST, with values similar or even higher than those observed during the first hospitalization (6th DPS of YF), indicating a high and persistent liver cell damage. In contrast to two previous cases of relapsing to hepatitis two months after the onset of YF [4], the patient described here was symptomatic and also had a rebound in total and direct bilirubin and jaundice. When the patient was rehospitalized, we performed some tests to investigate the cause of the late-relapsing hepatitis. Laboratory tests discarded the occurrence of autoimmune hepatitis, inflammatory liver diseases, metabolic liver disease, or new infections. We were able to analyze a liver biopsy (collected at the 93rd DPS of YF) and detected YFV antigens and wild-type YFV RNA. Unfortunately, we were not able to acquire full YFV genome sequences or to run tests to detect infectious particles in the liver biopsy, due to the scarcity of biological samples. Nevertheless, the results so far suggested YFV persistence in the liver up to three months after the onset of YF. The absence of RNAmia (indicating the lack of viremia), and the presence of anti-YFV IgM and IgG from the 8th day of the first symptoms forward are consistent with the expected pattern for YF, with IgM appearing after one week of disease onset [1] and IgG levels rising a few days later [13]. 

The analyzis of serum chemokines, cytokines, and growth factors suggested a massive increase in many soluble mediators at the 36th DPS onset and a relevant reduction in the majority of biomarkers in the latter phase (at the same time that transaminases and bilirubin levels reached normality). These results may indicate that altered profiles of circulating inflammatory regulatory mediators are associated with this outcome.

Our data, together with previous studies [4,14], reinforce the occurrence of late-relapsing hepatitis after YFV infection. Here, this clinical picture was concomitant to the rebound of serum transaminases and bilirubin; the reappearance of jaundice; the persistence of YFV antigens and YFV RNA in the liver; and histopathological features indicating continued liver cell damage. Despite the rebound, transaminases and direct bilirubin remained elevated for at least for 124 days after the YF symptoms onset. Furthermore, the arresting of hyporexia, asthenia, and adynamia was reported for at least 197 days after YF onset. These results bring new data on the clinical course of YF, and highlight the need for an extended patient follow-up, specifically regarding liver supportive care and the monitoring of hepatic function. Further studies to investigate the role of possible viral persistence and immune responses causing the late-relapsing hepatitis after yellow fever are needed.

## Figures and Tables

**Table 1:** Time-course of the clinical picture during acute yellow fever and late-relapsing hepatitis after yellow fever.

Timeline Month/DPS		Laboratory Tests (Followed by Normal Range Values/unit) #									
		AST	ALT	TBil	DBil	APhos	GGT	INR	Urea	Cr	CK
		10–40 U/L	10–40 U/L	0.3–1.0 mg/dL	0.1–0.3 mg/dL	30–120 U/L	9–50 U/L *		8–20 mg/dL	0.7–1.30 mg/dL *	55–170 U/L *
	4	4560	3710	na	na	na	na	na	118	2.17	na
	7 **^A^**	781	999	9.8	8.9	139	495	1	85.1	2	42
	8 **^B^**	537	674	9.5	8.4	144	855	0.88	47	1.3	na
Jan	9	434	571	9.1	8	154	9399	na	53.6	1.4	na
	10	375	508	8.3	7.2	145	1057	1	39.1	1.2	38
	11	297	456	6.7	5.8	159	1103	na	na	na	na
	12	235	401	5.2	4.4	173	1074	na	40.2	1.4	na
	14 **^C^**	253	371	5.1	4.2	149	869	1	39.7	1.2	na
Feb	36 **^D^**	233	353	1.5	0.9	147	530	1.02	33.5	1.5	na
	64	677	797	10.4	6.5	142	355	1	44	1.3	na
	70	655	819	13.9	7	485	401	1.06	51	1.2	na
Mar	78 **^E^**	744	862	7.2	6.2	210	431	1.03	43.5	1.4	na
	80 **^F^**	660	1007	6.5	5.5	164	624	1	na	na	na
	82 **^G^**	793	973	6.4	5.5	163	447	1.24	na	na	na
	83 **^H^**	903	1235	6.1	5.2	171	572	1	na	na	na
	85 **^I^**	987	1285	5.7	4.9	182	448	1	na	na	na
Apr	87	1002	1666	6.5	5.4	193	444	1	na	na	na
	89 **^J^**	883	1252	5.9	4.9	192	525	1	48	1.4	na
	92	769	904	3.8	3.3	207	554	1	51.4	1.4	na
	93	na	na	na	na	na	na	na	na	na	na
	95	743	863	3.6	3.1	229	547	na	45	1.3	na
May	124	78	108	1.3	1	139	199	1	34.9	1.2	na
Jul	186	21	28	0.84	0.2	na	71	na	44	1.05	na
	197 **^K^**	27	39	1	0.4	65	72	1	43.1	1.2	na
Nov	306 **^L^**	22	26	0.8	0.2	72	na	1.12	37.1	1.1	na

DPS: day after the first symptoms of yellow fever onset. na: not available. AST: aspartate aminotransferase, ALT: alanine aminotransferase, APhos: alkaline phosphatase, GGT: gamma-glutamyltransferase, TBil: total bilirubin, DBil: direct bilirubin, Cr: Creatinine, CK: Creatine kinase, INR: international normalized ratio (INR) for prothrombin time. The days when the patient was hospitalized are shown in blue, the periods when the patient had elevated AST and ALT are shown in green and periods when the patient had jaundice are in orange. **A**: anti-HIV-1 and anti-HIV-2 non-reactant. **B**: anti-YFV IgM and IgG reagent; anti-DENV IgM non-reactant. **C**: anti-YFV IgM reagent. Hospital discharge. **D**: anti-YFV IgG reagent. **E**: Serum and urine were tested by qPCR for YFV [5] and results were negative. Serum was tested by qPCR and negative for DENV [6,7], CHIKV [8], and pan-flaviviruses [9]. Anti-YFV IgM and IgG reagent. Neutralizing antibodies anti-YFV were detected by PRNT_80_ (up to sera dilution 1:80) [10]. Patient was rehospitalized and computed tomography indicated mild hepatomegaly but no biliary obstruction. **F**: Non reactant tests for anti-smooth muscle, anti-mitochondrial, anti-nuclear factor HEP2, anti-neutrophil cytoplasm antibodies (P-ANCA and C-ANCA). Total IgG = 962 mg/dL (6417.6 µmol/L) (normal range^11^: 800–1500 mg/dL), erythrocyte sedimentation rate = 41 mm/hour (normal range for male^11^: 0–15 mm/hour), serum copper = 138 µg/dL (21.72 µmol/L) (normal range^11^ = 100–200 μg/dL), tree T4 = 0.98 ng/dL (12.61 pmol/L) (normal range^11^: 0.8–1.8 ng/dL), thyroid-stimulating hormone = 2.53 µU/mL (normal range^11^: 0.5–4.0 μU/mL), ferritin 7.46 ng/mL (16.76 µg/L) (normal range for male^11^: 24–336 ng/mL), and ceruloplasmin = 29.1 mg/dL (2.17 µmol/L) (normal range^11^: 25–43 mg/dL). **G**: Non-reactant tests for: anti HIV-1 and HIV-2; anti-HCV; anti-HAV IgM and IgG; anti-Epstein Barr virus IgM and IgG; anti-toxoplasma IgM. Reagent tests for anti-toxoplasma IgG. **H**: Anti-YFV IgM and IgG reagent. Neutralizing antibodies anti-YFV were detected by PRNT_80_ (up to sera dilution 1:80) [10]. **I**: Ultrasonography indicated non-specific increased periportal echogenicity and mild hepatomegaly. **J**: Tests were run and non-reactant for IgM anti-HBc, total anti-HBc, and HBsAg. Colangio magnetic resonance indicated mild splenomegaly. **K**: anti-YFV IgG reagent. **L**: anti-YFV IgM and IgG reagent. # normal range values are presented according to American Board of Internal Medicine Laboratory Test Reference Ranges—January 2020 [11]. * normal range values for males. Normal range are presented in International System Units (SI), calculated by the multiplication of values in conventional units per conversion factor (CF), as follows: AST: 10–40 UL × 0.0166 (CF) = 0.17–0.66 microkatal units/L, ALT: AST: 10–40 UL × 0.0166 (CF) = 0.17–0.66 microkatal units/L, TBil: 0.3–1.0 mg/dL × 17.1 (CF) = 5.13–17.1 µmol/L, DBil: 0.1–0.3 mg/dL × 17.1 (CF) = 1.71–5.13 µmol/L, AP: 30–120 U/L × 0.01667 (CF) = 0.5–2.0 microkatal units/L, GGT: 9–50 U/L × 0.016667 (CF) = 0.15–0.83 microkatal units/L, Urea: 8–20 mg/dL × 357.146 (CF) = 2857.14–7142.86 µmol/L, Creatinine: 0.7–1.30 × 88.42 (CF) = 61.89–114.95 µmol/L, CK: 55–170 U/L × 0.01667 (CF) = 0.92–2.83 microkatal units/L, total IgG:800–1500 mg/dL × 6.67 (CF) = 5336.9–10,006.7 µmol/L, ferritin: 24–336 ng/mL × 1.0 (CF) = 24–336 µgl/L, free T4 = 0.8–1.8 ng/dL × 12.86 (CF) = 10.29–23.16 pmol/L, ceruloplasmin = 25–43 mg/dL × 0.6667 (CF) = 1.86–3.20 µmol/L, serum copper: 100–200 μg/dL × 0.157 (CF) = 15.74–31.47 µmol/L).

## Supplementary Materials

The following are available online at https://www.mdpi.com/1999-4915/12/2/222/s1. File S1: Supplementary methods. Table S1: Hemoglobin and blood cells counts during acute yellow fever and late-relapsing hepatitis after yellow fever. Figure S1: Serum levels of anti-YFV IgG.
