# Supplementary material for: Late-Relapsing Hepatitis after Yellow Fever
Source: Viruses. 2020 Feb 17;12(2):222. doi: 10.3390/v12020222 (PMC7077229; doi:10.3390/v12020222)
Supplement: Supplementary file 1 [file viruses-12-00222-s001.zip › viruses-688701.suppl zip/sup material/viruses-688701-File S1.pdf]

Supplementary Methods

Clinical markers

Routine tests for clinical parameters were performed in hospitals where the patient was admitted. In Eduardo de Menezes Hospital (HEM), exams to measure AST, ALT, and APhos were run by the multipoint kinetic method. Exams for measuring of GGT and CK were run by enzyme/colorimetric method. Levels of Cr were estimated by the two-point kinetic method, while levels of the TBil and DBil were determined by the colourimetric method. Exams to evaluate blood cell counts (leukocytes, neutrophils, lymphocytes, hematocrit, and haemoglobin) were run by automated counting through flow cytometry. Markers for autoimmune hepatitis and metabolic liver disease were run: anti-smooth muscle, anti-mitochondrial, anti-nuclear factor HEP2, anti-neutrophil cytoplasm antibodies (by the indirect immunofluorescence method), serum Copper (enzymatic method), total IgG, and ceruloplasmin (immunoturbidimetry method).

Diagnosis of infectious diseases

Routine laboratory tests for diagnosis of infectious diseases were performed in Eduardo de Menezes Hospital or at the State Reference Laboratory at Fundação Ezequiel Dias, Minas Gerais. Antibodies against HIV-1 and HIV-2 were investigated using immunochromatography tests (Genie Fast HIV 1/2 Assay - Bio-Rad Genie™, Hercules, CA, USA). HBsAg and antibodies anti-HCV, anti-HAV (IgM and IgG), anti-Epstein Barr virus (IgM and IgG), total anti-HBc, anti-toxoplasma (IgM and IgG) were investigated by chemiluminescence method, and anti-HBc (IgM) were investigated by electrochemiluminescence method, using routine tests implemented in HEM. IgM antibodies against yellow fever virus (anti-YFV IgM) and IgM antibodies against dengue virus (anti-DENV IgM) were investigated using in house tests (MAC-ELISA), performed by the State Reference Laboratory at Fundação Ezequiel Dias, Minas Gerais.

Serological, immunological, and virological investigation of the patient with relapsing hepatitis following yellow fever (YF)

Tests to investigate the presence of YFV RNA, antibodies against YFV, and the immunological response of the patient were run in the Laboratory of Virus/Universidade Federal de Minas Gerais and Instituto René Rachou/Fundação Oswaldo Cruz, Minas Gerais, Brazil.

The analysis of serum biomarkers of the immune response was carried out in blood samples collected at the 36th, 78th, 197th, and 306th day post symptom onset (DPS). The levels of chemokines (CXCL8, CCL11, CCL3, CCL4, CCL2, CCL-5, and CXCL10), pro-inflammatory cytokines (IL1- β, IL-6, TNF-α, IL-12, IFN-γ, IL-15 and IL-17), regulatory cytokines (IL-1Ra, IL-4, IL-5, IL-9, IL-10, and IL-13) and growth factors (FGF-basic, PDGF, VEGF, G-CSF and GM-CSF, IL-2 and IL-7) were measured, using a high-performance microbeads 27-plex assay, according to the manufacturer's instructions (Bio-Rad, Hercules, CA, USA). The biomarker levels detected in the YF patient were compared to those observed in age-matched non-infected healthy controls (n=16). The value observed for each biomarker was compared with the 95% confidence interval (95%CI) of the mean values found in the reference control group.

IgM antibodies (anti-YFV IgM) were investigated using different sera (collected at the 8th, 78th, 83rd, and 306th DPS) by immunochromatography test, according to the manufacturer's instructions (EcoDiagnóstica, Brazil: Febre Amarela IgM ECO Teste - TR.0040C: sensitivity: 99,5% and specificity: 98%).

IgG antibodies against YFV were investigated using sera collected at 8th, 36th, 78th, 83rd, 197th, and 306th DPS by in-house ELISA. 96 wells plate was coated with 2.5 μg/mL of YFV-17DD diluted in 50 μL/well of coating buffer (carbonate-bicarbonate buffer pH 9.6) and incubated overnight at 4°C. After, the plate was mechanically washed five times with 300 μL/well using washing buffer (PBS pH 7.4 with 0.05% Tween-20 –PBS/T). All afterwards washing steps were done as described above. The plate was then blocked with 100 μL/well using blocking/diluent solution (BDS) (PBS/T, 0.05% BSA, 3% fetal bovine serum (FBS) and 5% skimmed milk), for 1 h at 37 °C. The sera samples were diluted 1:20 and then serially two-fold diluted up to 1:160. For the standard curve, antibody Anti-Yellow Fever Serum (YF - NIBSC) was diluted in two-fold serial dilutions using BDS, ranging from 1 to 0.015 UI/mL. After 1 hour at room temperature (RT), the plate was washed and incubated with 100 μL/well with commercial antibody HRP Anti-Human IgG (BD Bioscience) diluted 1:3000 in BDS and incubated for 1 hour at RT. The plate was washed, and 100 μL/well of substrate solution (TMB plus TM) was added. The plate was incubated for 15 min at room temperature and, after that, 100 uL/well of stop-solution (2M H_2SO_4) was added. The endpoint measurements were done at 450 nm using spectrometer. The absorbances of serum sample dilutions were plotted on the standard curve. The antibody titers were calculated using the software SoftMax Pro® by regression logistic for four parameters and expressed in IU/mL, relative to the reference antiserum.

Investigation of neutralising antibodies against YFV was performed by plaque neutralisation assay [1], using samples collected at the 8th, 78th, and 83rd. Sera dilutions from 1:20 to 1:640 were used [1], and samples with a reduction of at least 80% of viral plaques, compared to the virus control (vaccine strain YFV-17DD), were considered positive for the presence of YFV neutralizing antibodies [10].

Urine and sera (collected at the 78th DPS) were tested for the presence of RNA belonging to YFV, DENV, Chikungunya virus (CHIKV). Briefly, 140μl of serum or urine was used for total RNA extraction using QIAmp Viral RNA Mini Kit (Qiagen, USA) following the manufacturer's instructions. RNA samples were reversed transcribed using 14μl of RNA and M-MLV reverse transcriptase kit (Promega) following the manufacturer's instructions. A total of 2.5 μl of cDNA was used as a template in a real-time polymerase chain reaction, using primers targeting regions within the genome of YFV[5] DENV[6,7], CHIKV[8] or pan-flaviviruses primers[9].

The liver biopsy collected at 93rd DPS, was used for histological analysis. A fragment of liver biopsy was fixed in 10% neutral buffered formalin, followed by paraffin embedding, micron-thick sectioned and staining by hematoxylin and eosin (H&E) method. For immunohistochemistry analysis, we used anti-YF primary antibody and protocol that were produced and standardised at the Evandro Chagas Institute (anti-YFV monoclonal antibody was kindly provided by Dr. Pedro Fernando da Costa Vasconcelos, from Evandro Chagas Institute, Pará, Brazil).

Following, we investigated the presence of YFV RNA in the liver biopsy. The liver biopsy (9 mg) was used for total RNA extraction, using RNeasy Minikit (Qiagen, USA) following the manufacturer's instructions. RNA was reversed transcribed using 14.0 μl of RNA and M-MLV reverse transcriptase kit (Promega) following the manufacturer's instructions. A total of 2.5 μl of cDNA was used as a template in a real-time polymerase chain reaction, using primers targeting the YFV 5′-UTR [5]. Next, we performed a quantitative PCR using the kit Bio Gene Research Febre Amarela PCR (Bioclin, Brazil. Sensitivity: 2 YFV genomic copies/reaction). A standard quantification curve was composed of serial ten-fold dilutions of positive control (2.0 x 10^5 to 2.0 genomic copies/μl). We estimated the number of genomic copies per gram (gc/g) of liver biopsy according to kit instructions, using as input the amount of liver (grams) used for RNA extraction, and with proper adjustments regarding the dilution factor. After the detection of YFV RNA in liver biopsy, another qPCR was performed targeting partial NS5 nucleotide sequence of flaviviruses [9]. The amplified DNA (261 nucleotides) was purified and sequenced by the dideoxy method on an ABI3130 platform (Applied Biosystems). Raw data was analysed,

and the final contig was assembled using Geneious v 9.1.8 (https://www.geneious.com). A phylogenetic tree was reconstructed using 60 YFV sequences retrieved from GenBank (www.ncbi.nlm.nih.gov/genbank). The sequences were aligned using Clustal W, implemented in MEGA7 [12]. The Kimura 2-parameters nucleotide substitution model with a gamma distribution (four categories) was used for the reconstruction of trees using the maximum likelihood method with 1000 bootstrap replicates, using MEGA7 [12]. Genbank accession numbers of sequences used for phylogenetic reconstruction were: AF094612, AY572535, AY603338, AY640589, AY968064, AY968065, DQ235229, GQ379162, GQ379163, HM582851, JF912179, JF912180, JF912181, JF912182, JF912183, JF912184, JF912185, JF912186, JF912187, JF912188, JF912190, JN620362, JX898868, JX898869, JX898870, JX898871, JX898872, JX898874, JX898875, JX898876, JX898877, JX898878, JX898879, JX898880, JX898881, KF769015, KF769016, KF907504, KM388814, KM388815, KM388816, KM388817, KM388818, KU921608, KU949599, KU978763, KU978764, KU978765, KX010994, KX010995, KX010996, KX027336, KX268355, KX982182, KY587416, KY861728, KY885000, KY885001, U21056, U54798.

References

5. Domingo, C.; Patel, P.; Yillah, J.; Weidmann, M.; Mendez, J.A.; Nakoune, E.R.; Niedrig, M. Advanced Yellow Fever Virus Genome Detection in Point-of-Care Facilities and Reference Laboratories. *J. Clin. Microbiol.* **2012**, *50*, 4054–4060.
6. Alm, E.; Lindegren, G.; Falk, K.I.; Lagerqvist, N. One-step real-time RT-PCR assays for serotyping dengue virus in clinical samples. *BMC Infect. Dis.* **2015**, *15*, 493.
7. Santiago, G.A.; Vergne, E.; Quiles, Y.; Cosme, J.; Vazquez, J.; Medina, J.F.; Medina, F.; Colón, C.; Margolis, H.; Muñoz-Jordán, J.L. Analytical and Clinical Performance of the CDC Real Time RT-PCR Assay for Detection and Typing of Dengue Virus. *PLoS Negl. Trop. Dis.* **2013**, *7*, e2311.
8. Kar, P.K.; Nagpal, B.N.; Dua, V.K.; Ghosh, S.K.; Raghavendra, K.; Bhatt, R.M.; Anvikar, A.; Das, A. Molecular characterization of chikungunya virus from Andhra Pradesh, India. *Indian J. Med. Res.* **2009**, *129*, 335–7.
9. Patel, P.; Landt, O.; Kaiser, M.; Faye, O.; Koppe, T.; Lass, U.; Sall, A.A.; Niedrig, M. Development of one-step quantitative reverse transcription PCR for the rapid detection of flaviviruses. *Virol. J.* **2013**, *10*, 58.
10. Wieten, R.W.; Jonker, E.F.F.; Pieren, D.K.J.; Hodiamont, C.J.; van Thiel, P.P.A.M.; van Gorp, E.C.M.; de Visser, A.W.; Grobusch, M.P.; Visser, L.G.; Goorhuis, A. Comparison of the PRNT and an immune fluorescence assay in yellow fever vaccinees receiving immunosuppressive medication. *Vaccine* **2016**, *34*, 1247–1251.
12. Kumar, S.; Stecher, G.; Tamura, K. MEGA7: Molecular Evolutionary Genetics Analysis Version 7.0 for Bigger Datasets. *Mol. Biol. Evol.* **2016**, *33*, 1870–1874.
