# Supplementary material for: Late-Relapsing Hepatitis after Yellow Fever
Source: Viruses. 2020 Feb 17;12(2):222. doi: 10.3390/v12020222 (PMC7077229; doi:10.3390/v12020222)
Supplement: Supplementary file 1 [file viruses-12-00222-s001.zip › viruses-688701.suppl zip/sup material/viruses-688701-supplementary legend.pdf]

Supplementary Figure S1. Serum levels of anti-YFV IgG. IgG antibodies anti-YFV were investigated by in house ELISA, using different serum samples collected at the 8th, 36th, 78th, 83rd, 197th, and 306th day post-symptoms onset (DPS). The antibody titers were calculated using the software SoftMax Pro® by regression logistic for four parameters and expressed in IU/mL relative to the reference antiserum, as described in supplementary methods.
