# Supplementary material for: Late-Relapsing Hepatitis after Yellow Fever
Source: Viruses. 2020 Feb 17;12(2):222. doi: 10.3390/v12020222 (PMC7077229; doi:10.3390/v12020222)
Supplement: Supplementary file 1 [file viruses-12-00222-s001.zip › viruses-688701.suppl zip/sup material/viruses-688701-Table S1.pdf]

Table S1: Hemoglobin and blood cells counts during acute yellow fever and late relapsing hepatitis after yellow fever

Timeline Month/DPS		Laboratory tests (normal range values /unit)#					
		Platelets (150,000-450,000mm³)	Hemoglobin (14-18 g/dL*)	Hematocrit (42 - 50%*)	Leukocyte (4,000 - 11,000 mm³)	Neutrophil (2,000 - 8,250 mm³)	Lymphocyte (30% - 45%)
Jan	4	73,000	17	50	4250	2,550	34.0
	7	88,000	13.7	41.8	2,900	1,711	21.0
	8	136,000	13.1	40.2	4,800	2,688	28.0
	9	142,000	12.6	na	4,700	na	na
	10	143,000	12.3	38.1	4,600	2,944	31.3
	11	152,000	11.8	36.7	4,400	2,860	27.0
	12	174,000	11.8	36.5	5,500	3,850	24.0
	14	178,000	12.5	39.6	6,300	4,473	21.0
Feb	36	158,000	11.6	34.8	4,700	2820	29.0
Mar	64	171,000	14.2	na	na	na	na
	70	189,000	14.8	na	na	na	na
	78	189,000	13.4	39.3	4,900	3,479	25.0
	82	144,000	12.9	38.5	5,100	2,805	35.0
Apr	89	153,000	14	41.1	7,400	5,180	20.0
	92	131,000	12.2	35.1	3,400	1,938	31.0
	93	105,000	13.5	38.8	3,700	2,183	32.0
	95	164,000	13.2	38.6	4,900	2,989	30.0
May	124	185,000	13.3	37.9	7,000	4,690	26.0
Jul	186	150,000	16.5	46.8	7,280	na	28.8
	197	159,000	17.7	52.3	7,600	5,092	26.0
Nov	306	145,000	17.4	49.9	7,000	4,760	25.0

DPS: day post symptom onset of yellow fever. na: not available. The days when the patient was hospitalized are shown in blue, the periods when the patient had elevated levels of aspartate transaminase (AST) and alanine transaminase (ALT) are shown in green, and periods when the patient had jaundice are shown in orange. #normal range values are presented according to American Board of Internal Medicine Laboratory Test Reference Ranges—January 2020[11]. Normal range are presented in International System Units (SI), calculated by the multiplication of values in conventional units per conversion factor (CF), as follows: platelet: 150,000-450,000 mm³ x 0.001 (CF) = 150-450 x 10^9/L, hematrocrit: 41-53% x 0.01 (CF) = 0.41-0.53 fraction, leukocyte: 3,500 - 10,000 mm³ x 0.001 (CF) = 3.5 -10 x 10^9 cell/L, and neutrophil: 1,600 – 8,000 mm³ x 1.0 (CF) = 1,600 – 8,000 cell/μL.
