# Supplementary figures and images for: Late-Relapsing Hepatitis after Yellow Fever
Source: Viruses. 2020 Feb 17;12(2):222. doi: 10.3390/v12020222 (PMC7077229; doi:10.3390/v12020222)

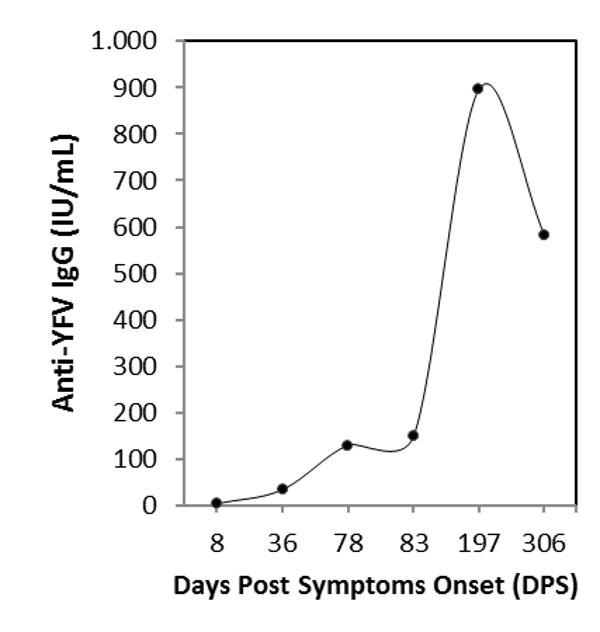

Supplement: Supplementary file 1 [file viruses-12-00222-s001.zip › viruses-688701.suppl zip/sup material/Figure S1.tiff]
